# Patient Profile and Cost Savings of Long-Term Care in a Spanish Hospital: Retrospective Observational Study

**DOI:** 10.2196/64248

**Published:** 2024-11-19

**Authors:** José Joaquín Mira, Daniel García-Torres, María del Mar Bonell-Guerrero, Ana Isabel Cáceres-Sevilla, Martina Ramirez-Sanz, Rosa Martínez-Lleo, Concepción Carratalá

**Affiliations:** 1 Health Psychology Department Universidad Miguel Hernández Elche Spain; 2 Foundation for the Promotion of Health and Biomedical Research of the Valencian Community (FISABIO) Alicante Spain; 3 Hospital Padre Jofre Valencia Spain

**Keywords:** chronicity, length of stay, hospital, chronic, long-term care, demographics, gerontology, Hospitals for Acute and Chronic Long-Term Extended Stay, HACLES, healthcare economics, cost savings

## Abstract

**Background:**

Long-term care hospitals have been considered an efficient response to the health care needs of an increasingly aging population. These centers are expected to contribute to better hospital bed management and more personalized care for patients needing continuous care. The evaluation of their outcomes is necessary after a sufficient period to assess their impact. Hospitals for Acute and Chronic Long-Term Extended Stay (HACLES) emerged in Spain in the late 20th century as a response to the aging population and the increase in chronic diseases.

**Objective:**

This study aimed to analyze the profile of patients treated in a HACLES, particularly analyzing gender differences, and evaluate the cost savings associated with using these centers.

**Methods:**

A retrospective study was conducted based on data from patients 65 years old or older admitted to a HACLES between 2022 and 2023. Gender, age, household cohabitation data, diagnosis and comorbidity, daily medication intake, and degree of dependency were obtained to describe the profile of patients who attended the HACLES. Data coded in SIA-Abucasis (version 37.00.03; Consellería Sanitat, Generalitat Valenciana; a digital medical record system used in the Valencian region) were reviewed, and descriptive statistics and comparison tests were used. The direct cost savings of HACLES admissions were calculated by comparing the daily cost of a general hospital bed with that of a HACLES bed.

**Results:**

Data from 123 patients with a mean age of 77 years were analyzed. Most (n=81, 65.9%) had a cohabiting family member as their primary caregiver. Palliative care was the most frequent reason for admission (n=75, 61%). The mortality rate (odds ratio [OR] 61.8, 95% CI 53.2-70.5) was similar between men and women (OR 54.1, 95% CI 47.8-71.5 vs OR 59.7, 95% CI 42.2-66.0; *P*=.23). The cognitive assessment, using the Pfeiffer scale, improved at discharge (mean 3.2, SD 3.2 vs mean 2.5, SD 3.1; *P*=.003). The length of stay was significantly larger for patients who returned home compared with patients discharged to other facilities (mean 89.8, SD 58.2 versus mean 33.1, SD 43.1 days; *P*<.001). The direct cost savings were estimated at US $42,614,846 per 1000 admissions.

**Conclusions:**

Patients typically treated in HACLES are older, with a high level of cognitive impairment and physical dependency, and a significant proportion are in palliative care, highlighting the importance of adapting care to the individual needs of the admitted patients. The HACLES model contributes to the sustainability of the public health system.

## Introduction

In all high-income countries, long-term care hospitals have been considered an efficient response to the health care needs of an increasingly aging population that have multiple chronic conditions simultaneously [1]. However, the care model is being reviewed, and new ideas are emerging to leverage the potential of these health care resources [2].

In Spain, Hospitals for Acute and Chronic Long-Term Extended Stay (HACLES) were initially designed to free up beds in general hospitals occupied by patients with chronic conditions who did not require an acute care but needed a suitable hospital environment [3]. These hospitals continue to focus on managing complex medical needs that extend beyond short-term acute care settings, with an emphasis on long-term care and support for patients with chronic illnesses, disabilities, or conditions requiring prolonged medical treatment.

In the mid-90s, HACLES were established in the Valencian Community as a response to the need for specialized care for patients with chronic conditions and those requiring long-term care. These centers integrated medical, nursing, rehabilitation, psychological, and social support [4]. By the early 21st century, this model was consolidated with investments to make them more accessible and comfortable for prolonged stays, adapting to the profiles and needs of patients with chronic conditions, including those requiring palliative care. These centers are expected to contribute to better hospital bed management and more personalized care for patients needing continuous care.

The HACLES model, as is happening in other countries [5] and having gone through its period of implosion and consolidation, requires the development of long-term strategies that respond to the demographic and technological changes in our society. This study aims to describe the profile of patients receiving care in a HACLES, particularly analyzing gender differences, and evaluate the cost savings associated with using these centers.

## Methods

### Overview

A retrospective observational study was conducted based on the review of data coded in SIA-Abucasis (the digital clinical history system used in the Valencian Community) from a systematic sample of patients >65 years old admitted to the HACLES. The STROBE (Strengthening the Reporting of Observational Studies in Epidemiology) guidelines were used to describe this study (Multimedia Appendix 1).

### Patient Eligible Criteria

Data were recorded from all patients admitted over a period 2 years, between January 2022 and December 2023. A blinded registration system was used for the research team. A web-based platform was developed to ensure data quality, accessed through a personalized key, respecting data anonymization.

### Sample Size

Considering the annual number of admissions (N=294), an α risk of .05, and a precision of 5%, the sample size was estimated at 168 medical records. This calculation was made using the formula for sample size in finite populations.

### Sample Selection

Among the patients randomly assigned to each reviewer, a total of 30 cases were selected through simple randomization (k=3).

The reviewers included 4 nurses (all women). The reviewers were trained in the review procedure and in using a data registration tool, ensuring uniformity in the interpretation of the study protocol. A call center was available during the field study to resolve any issues related to data entry on the web platform.

### Study Variables

These included binary variables such as gender; continuous variables such as age, polypharmacy (daily medication intake), average stay, and, when available, Barthel [6], Pfeiffer [7], and Gijón [8] scales scores at admission and discharge; and nominal variables such as household cohabitation data, primary diagnosis, comorbidity (based on the *International Classification of Diseases, 10th revision* [*ICD-10*]), reason for admission, degree of dependency, residence at the time of admission, destination upon discharge (home, institution, death, or others), and who the primary caregiver is.

### Cost Analysis

The direct cost savings represented by admission to a HACLES were estimated by the difference between the daily cost of a bed in a general hospital, established at US $1047 [9], and the daily cost of a bed in a HACLES, estimated at US $373 [10]. Declared costs were adjusted to 2024 values considering increases in the cost of living.

### Data Analysis

Data curation included eliminating outliers in the variable of hospital stay duration to avoid distorting the used statistics. Specifically, outliers in the variable of hospital stay duration were identified using the IQR method. Observations falling below the first quartile minus 1.5 times the IQR or above the third quartile plus 1.5 times the IQR were considered outliers. These were removed to prevent distortion in the statistical analyses, ensuring a more accurate reflection of the central tendency and variability.

Descriptive statistics (mean and SD) were used to summarize data and identify patterns. Comparative analyses were conducted using both parametric and nonparametric tests, depending on the data distribution. The Student *t* test (2-tailed) was used to compare men and women for continuous variables, while the chi-square test was used for qualitative variables such as identifying patterns in the origin and destination of patients after discharge. The Cochran-Mantel-Haenszel test was conducted to analyze gender-adjusted differences in assistance rates. Nonparametric tests such as Kruskal-Wallis and Wilcoxon tests were used when assumptions for parametric tests were not met. Statistical significance was set at *P*<.05 (2-sided) for all tests.

### Ethical Considerations

The Ethics Committee of the Health Department of Alicante-Sant Joan approved the study (reference 24/044). The Ethics Committee waived the requirement to obtain informed consent from all patients, as it was an epidemiological study that met the criteria of necessity, proportionality, and adequacy, offering comprehensive guarantees for the protection of personal data and respect for the privacy of the individuals involved, in accordance with Spanish Law 14/2007 on Biomedical Research.

## Results

Data from a total of 123 patients (73.2% of the expected total of 168) were recorded. Almost half of the sample were men (61/123, 49.6%). The mean age of the sample was 77.2 (SD 13.1) years. In 81 (65.9%) cases, the primary caregiver was a cohabiting family member (Table 1).

The average duration of the hospital stay was 83.3 (SD 80.5) days. Excluding outliers, the stay for men (55.2, SD 52.6 days) and women (53.1, SD 53.4 days) was similar (*P*=.21). At discharge, the number of daily medications administered to patients was 7.7 (SD 4) for men and 8.2 (SD 3.8) for women (*P*=.33). The reason for admission to the HACLES was similar between men (36/61, 59% palliative care; 14/61, 23% rehabilitation; 5/61, 8.2% convalescence; and 6/61, 9.8% long stay) and women (39/62, 62.9% palliative care; 14/62, 22.6% rehabilitation; 5/62, 11.3% convalescence; and 6/62, 3.2% long stay; *P*=.52).

**Table 1:** Sample description.

Variable			Total (N=123)		Men (n=61, 49.6%)		Women (n=62, 50.4%)
**Age (years), mean (SD)**			77.2 (13.1)		79.2 (12.8)		74 (11.8)
**BMI (kg/m^2^), mean (SD)**			23.9 (5.4)		22.8 (4.8)		25.4 (6.0)
**Primary caregiver (multiple options could be selected), n (%)**							
	Institution	2 (1.6)		0 (0)		2 (2.8)	
	Cohabiting caregiver	81 (65.9)		42 (58.3)		39 (58.3)	
	Hired caregiver	21 (17.1)		10 (13.9)		11 (15.3)	
	Self (loneliness)	7 (5.7)		3 (4.2)		4 (5.6)	
	No identified caregiver	9 (7.3)		4 (5.6)		5 (6.9)	
	Other (eg, supervision by descendants, support from a nongovernmental organization, etc)	21 (17.1)		10 (13.9)		11 (15.3)	
**Emergency contact, n (%)**							
	Spouse	44 (35.8)		28 (45.9)		16 (25.8)	
	Child	54 (43.9)		19 (31.1)		35 (56.4)	
	Sibling	13 (10.6)		7 (11.5)		6 (9.7)	
	Other family	12 (9.8)		7 (11.5)		5 (8.1)	
**Degree of dependency, n (%)**							
	None	57 (46.3)		28 (45.9)		29 (46.8)	
	Moderate	0 (0)		0 (0)		0 (0)	
	Severe	9 (7.3)		4 (6.6)		5 (8.1)	
	High dependency	10 (8.1)		3 (4.9)		7 (11.3)	
	Under review	47 (38.2)		26 (42.6)		21 (33.9)	
**Receiving teleassistance, n (%)**			6 (4.9)		2 (3.3)		4 (6.5)
**Reason for admission, n (%)**							
	Palliative care	75 (61)		36 (59)		39 (62.9)	
	Rehabilitation	28 (22.8)		14 (23)		14 (22.6)	
	Convalescence	12 (9.8)		5 (8.2)		7 (11.3)	
	Long-term stay	8 (6.5)		6 (9.8)		2 (3.2)	
**Risk of falling at admission, mean (SD)**			73 (43.3)		40 (26.2)		33 (17.6)
**Number of chronic conditions at admission, mean (SD)**			5.3 (4.1)		4.8 (4.4)		5.5 (4.2)

In total, 70 patients died during their stay at the HACLES, while 54 were discharged and returned home (Table 2). The mortality rate was similar between men and women (men: 54.1%, 95% CI 47.8-71.5; women: 59.7%, 95% CI 42.2-66.0; *P*=.23). The degree of dependency at admission was higher in patients who eventually died compared with those who did not (*P*=.008; Table 2).

**Table 2:** Factors related to mortality.

Variable			Died during admission			*P* value	
		Yes (n=70)		No (n=53)			
**Scale, mean (SD)**							
	Pfeiffer at admission	4.3 (2.3)		3.3 (3.2)	.11		
	Barthel at admission	27.4 (33.4)		29.2 (33.2)	.75		
**Degree of dependency at admission^a^, n (%)**							.008^b^
	Moderate	0 (0)		0 (0)			
	Severe	4 (5.7)		5 (9.4)			
	High dependency	4 (5.7)		6 (11.3)			
	None	45 (64.3)		12 (22.6)			
**Reason for admission, n (%)**							.52
	Palliative care	39 (55.7)		36 (67.9)			
	Rehabilitation	14 (20)		14 (26.4)			
	Convalescence	7 (10)		5 (9.4)			
	Long-term stay	2 (2.9)		6 (11.3)			

^a^47 patients were under review and were not included.

^b^The chi-square test does not include patients under review.

The average length of stay for patients who died at the HACLES was 35.4 (SD 80.1) days for women and 48.4 (SD 44.8) days for men (*P*=.40).

A total of 57 (46.3%) of the 123 patients admitted to the HACLES had a nonfamily caregiver or were institutionalized. Among those cared for by a cohabiting family member (81/123, 65.9%), the recognized degree of administrative dependency was higher (mean 1.3, SD 1.1 vs mean 1.9, SD 0.7; *P*=.001).

At discharge, a higher number of women than men were referred to another residential institution (11/25, 44% vs 7/28, 25%), although the difference was not statistically significant (*P*=.16). Most returned to their home (men: 21/28, 75%; women: 13/25, 52%; *P*=.11; Table 3). The length of stay was significantly larger for patients who returned home compared with patients discharged to other facilities (mean 89.8, SD 56.8 vs mean 33.1, SD 36.9 days; *P*<.001).

**Table 3:** Origin and destination of patients treated in the HACLES (Hospitals for Acute and Chronic Long-Term Extended Stay).

Residence	At admission (N=123), n (%)	At discharge (N=123), n (%)	Men (n=61), n (%)	Women (n=62), n (%)
Home	113 (91.9)	34 (27.6)	21 (34.4)	13 (21)
Institution	2 (1.6)	18 (14.6)	7 (11.5)	11 (17.7)
Day center	5 (4.1)	1 (0.8)	0 (0)	1 (1.6)
Assisted living	1 (0.8)	0 (0)	0 (0)	0 (0)
Not recorded	2 (1.6)	0 (0)	0 (0)	0 (0)
Death	—^a^	70 (56.9)	33 (54.1)	37 (59.7)

^a^Not applicable.

Scores on the Barthel scale at discharge were 4 points higher than at admission, but this difference was not statistically significant (*P*=.46; Table 4). On the Pfeiffer scale, scores were higher at the beginning compared to discharge, with an initial score of 3.2 (SD 2.8) versus 2.5 (SD 2.8) at discharge (*P*=.03). At discharge, the Barthel score was the same for men and women (mean 36.4, SD 29.9 vs mean 30.2, SD 29.9; *P*=.45), while the Pfeiffer score for men was lower than for women (mean 1.7, SD 2.5 vs mean 3.9, SD 3.3; *P*=.01). The Gijón social-familial scale score was very similar for men and women at discharge (men: mean 7.6, SD 3.2; women: mean 8.3, SD 3.8; *P*=.27).

**Table 4:** Scores on the Barthel, Pfeiffer, and Gijón scales.

Scale at admission	At admission, mean (SD)	At discharge, mean (SD)	Men at discharge, mean (SD)	Women at discharge, mean (SD)
Barthel (autonomy)	29.7 (29.5)	33.5 (26.1)^a^	36.4 (29.9)	30.2 (29.9)
Pfeiffer (cognitive function)	3.2 (3.2)	2.5 (3.0)	1.7 (2.5)	3.9 (3.3)
Gijón (socio-familial support)	—^b^	8 (3.2)	7.6 (2.9)	8.3 (3.5)

^a^*P*=.04.

^b^Not applicable.

Table 5 summarizes the estimated cost savings represented by the HACLES, both for the study sample and the extrapolation per 1000 admitted patients, for both men and women, considering the average stay of this study. On average, for every 1000 patients admitted to HACLES, the total hospital stay cost is reduced by US $42,614,846 (considering 61/123, 49.6% men and 62/123, 50.4% women). If the average length of stay is not corrected by eliminating outliers (average stay of 98.6 days for male patients and 69 days for female patients calculated during the study period), the cost differential for admissions in a general hospital compared with HACLES would be US $56,877.01.

**Table 5:** Cost savings from admissions in HACLES (Hospitals for Acute and Chronic Long-Term Extended Stay) compared with a general hospital^a^.

	Men	Women
Average stay (days), mean (SD)	98.6 (119.9)	69.0 (82.7)
Estimated savings per 1000 patients (US $ millions)	46.3	39.8

^a^Average daily savings per bed of US $6280 compared with a general hospital.

## Discussion

### Principal Findings

The data from this study reflect that HACLES fulfill their function by offering an alternative admission option for patients requiring diversified and varying intensity care but for a longer duration, reducing the higher per-bed costs of general hospitals. The average age of patients admitted to a HACLES is around 77 years, with no significant differences between men and women. These patients have, on average, about 5 chronic conditions. In this sample, a significant proportion were admitted to the HACLES for palliative care, although almost a quarter were admitted for rehabilitation treatment in special conditions.

The average duration of stay in these centers is about 2 months, significantly different from the average of 7.5 days in general hospitals [11]. In this study, men had a 9.6-day longer stay than women, but this difference is not statistically significant due to the wide range of days of stay (including some stays up to 591 days).

HACLES could be responsible for saving over US $650 per patient per day of hospitalization, which means that both in terms of their specialization for the described patient profile and in economic terms, their existence is justified.

### Interpretation of Findings

Patients in long-term care facilities tend to be older and have a higher burden of chronic illnesses and comorbidities compared with the broader older adult population. Among the patients, 15 (15%) out of 100 had formal recognition of their level of dependency and were receiving state aid to manage their situation, with this being slightly more common among women than men. However, this does not imply that the patients admitted to HACLES had a less severe profile compared with other patients analyzed in different studies [12,13]; rather, it reflects the slow administrative process. Furthermore, it should be noted that almost 4 out of 10 patients admitted to HACLES are still awaiting evaluation of their degree of dependency due to significant delays in Spain’s evaluation, registration, and subsidy assignment process, which can extend for over a year and a half.

Slightly more than half of the patients admitted died, consistent with or slightly lower than findings reported in similar studies [12]. This result contrasts with the lower mortality rates reported in studies conducted in general hospitals [14] and among patients with severe cognitive decline admitted to critical care units [15].

Most of those who died did not have recognized dependency, although a part (27/70, 38.2%) was pending resolution or under study. The reason for admission was not related to the mortality rate. Among those with formal administrative recognition of dependency, this situation does not account for differences in mortality, length of stay, or discharge destination. The mortality rate in this study was practically the same between men and women, as highlighted in other studies conducted in contexts more comparable to this one [16].

Scores on the Barthel scale indicated that both men and women generally showed severe dependency. In this sample, women presented greater cognitive deterioration than men admitted during the study period. Greater functional impairment in women, along with a similar mortality rate between men and women, have been reported in other studies [17], and our data confirm this trend. In this case, while women’s social risk was moderate, men faced lower risk, which could explain their higher proportion of home returns upon discharge. However, it should be noted that the life expectancy for men (84.8 years) and women (88.3 years) in Spain [18] for this age group is significantly different.

The frequency with which women are referred to a residential institution upon discharge instead of returning home requires attention and new studies with larger samples. The trend in our data, combined with scores on autonomy, cognitive function, and social and family relationships, suggests that the difference may be due to women more frequently assuming the role of caregivers compared with men because of gender bias ingrained in our society. Other studies suggest that this is a phenomenon that extends beyond Spain [19]; thus, when planning alternative long-term care services, the different needs and preferences of men and women [19,20] should be taken into consideration.

The main reason for organizing care for this patient profile around a HACLES remains unchanged. Nearly 30 years after its implementation [21], its function is still necessary, and with the increase in chronic conditions, its capacity will likely be limited to respond to demand. The care needs required by older patients with multiple chronic conditions and limitations in their autonomy, along with the cost savings these centers have compared with general hospital admissions, corroborate the function of these centers, in line with what happens in other countries [22,23]. Furthermore, not only is the cost per bed lower but it is also important to consider, as some studies point out [24], that the number of medical tests and therapeutic interventions for this patient profile is higher when admitted to an acute care hospital. This increases risks for patients, affects their well-being, and raises the cost of care.

In the literature on long-term care hospitals and chronic patient management, the redistribution of spending across care settings has long been recognized usually as a decisive factor in cost savings [25,26]. The reinvestment of these savings should contribute to the expansion of services for older adults. Management models like the Chronic Care Model [27] have been adopted in various countries [28], including Spain [29], to provide a comprehensive approach to care. This aspect is crucial, as highlighted by the results of this study.

### Practical Implications

The health care teams in HACLES and their management teams could review their action protocols, promoting integrated actions with community resources to ensure a return home that addresses the social and personal needs of the people they serve, particularly in the case of women who show a greater tendency toward institutionalization after discharge. It should not be ruled out that while women commonly assume the role of informal caregivers for their male partners, men who take on this caregiving role face more limitations due to having less experience with domestic tasks. In this context, developing new approaches to involve relatives in long-term care is a significant challenge in many countries. Engaging family members effectively can improve patient outcomes and overall well-being, but it requires innovative strategies and resources to support both the patients and their caregivers [30].

Most patients were cared for by a cohabiting relative, typically the spouse or 1 or more children. Almost a quarter might present loneliness, which is in line with data analyzing the frequency of loneliness in the general population in Spain [31], despite almost half of this group receiving help from a non-cohabiting caregiver. This data suggest that one function HACLES could assume is to address, before discharge and in collaboration with community or social welfare resources, measures to limit the impact of loneliness upon return home. This would help address a growing social problem and reduce primary care costs [32].

The study findings have further implications for health care policies. First, since these facilities meet a significant practical need, particularly for older adult patients requiring prolonged hospital care at a much lower cost than general hospitals, health care planners should consider this approach as a viable alternative due to its efficiency in both public health systems, like Spain’s, and in other mixed or predominantly private systems. Second, health care planners and managers of health care structures, such as primary and community care, should work together to address the challenges of integrating care after discharge from a long-term care hospital, as has already been suggested [33]. Home care should be considered to maintain positive outcomes for as long as possible after leaving the long-term care facility, thereby preventing readmissions.

### Future Research

Given the high mortality rate within a year after discharge [16], it should be analyzed in relation to the discharge destination of these patients, monitoring differences between men and women and considering the different options available to each group.

The impact of HACLES activities diminishes if adequate postdischarge care is not ensured. This requires offering integrated care [28] that addresses the needs and preferences of both men and women during this phase of their lives. Further research providing insights into the critical success factors of these interventions would facilitate more rapid implementation of effective solutions when patients are discharged from long-term care facilities.

### Strengths

This study is conducted in a field where the number of studies is limited, particularly in Spain. It describes the profile of patients treated in HACLES, analyzing gender differences. It identifies potential gender bias in home care provision. It also analyzes cost differences with general hospitals.

### Limitations

This study was conducted in a HACLES in one Autonomous Community in Spain. There may be long-term care centers organized to treat patients with other chronic conditions that do not fit the profile of patients treated in this center. Information records in clinical histories may sometimes be incomplete. When interpreting these data, the impact of the long stay on postadmission mortality should be considered [34].

### Conclusion

In summary, HACLES contribute to the sustainability of the health care system, and the reasons for their creation remain valid. The profile of needs of the patients admitted to these centers requires an integral approach, combining clinical, rehabilitative, psychological, and social aspects, not only during admission but also to set up appropriate mechanisms and resources according to needs, to offer integrated quality care to this generally more vulnerable group.

## Appendix

Multimedia Appendix 1 STROBE (Strengthening the Reporting of Observational Studies in Epidemiology) checklist.

## Abbreviations

HACLES: Hospitals for Acute and Chronic Long-Term Extended Stay
ICD-10: International Classification of Diseases, 10th revision
OR: odds ratio
STROBE: Strengthening the Reporting of Observational Studies in Epidemiology

## Data Availability

The datasets generated during and/or analyzed during this study are available in the Open Science Framework repository [35].
